# The hunt for protective correlates of immunity to *Plasmodium falciparum* malaria

**DOI:** 10.1186/s12916-014-0134-1

**Published:** 2014-08-08

**Authors:** Ann M Moormann, V Ann Stewart

**Affiliations:** Program in Molecular Medicine, University of Massachusetts Medical School, Biotech 2, Suite 318, 373 Plantation Street, Worcester, Massachusetts 01605 USA; Preventive Medicine and Biometrics, Uniformed Services University of Health Sciences, 4301 Jones Bridge Road, Bethesda, Maryland 20814 USA

**Keywords:** Malaria, Functional antibody assay, Opsonic, Phagocytosis

## Abstract

Determining an immunologic correlate of protection against *Plasmodium falciparum* malaria has been the holy grail of natural infection studies, and sought after as an endpoint for malaria vaccine trials. An *in vitro* assay that provides an accurate and precise assessment of protective immunity to malaria would make smaller, short-duration studies feasible, rather than the currently powered study designs that use morbidity or mortality as outcomes. Such a biomarker would be especially desirable in situations where malaria control measures that result in decreases in clinical endpoints and putatively waning protective immunity have been implemented. In an article published in *BMC Medicine*, Osier and colleagues addressed this problem, and demonstrated that antibodies promoting opsonic phagocytosis of merozoites provide a functional link between antigen-specific responses and protection. Understanding the mechanisms conferring protection against malaria not only improves our knowledge of basic human immunology, but promises to help in the design of an effective malaria vaccine.

Please see related article: http://www.biomedcentral.com/content/pdf/1741-7015-12-108.pdf.

## Background

One of the reasons that malaria vaccine development is so challenging is the enormous complexity of the host–parasite interaction. The parasite itself has over 5,000 open reading frames, some of which are differentially expressed during the numerous distinct life-cycle stages within the human host. Even within the cycling erythrocyte stage that can create the febrile disease, the parasite expresses different antigens as an invading merozoite, as a growing trophozoite, and as an organizing schizont. This remarkable life-cycle complexity, coupled with an almost unequalled degree of antigenic variability within the global population of parasites, allows the parasite to evade many actions of the host’s immune system.

Thus, unlike many other infectious diseases, survival of one or more bouts of febrile malaria disease does not provide sterile protection against further disease or further infection. Although still only partially understood, repeated exposure does change the immune balance between the parasite and its host. Because children in endemic areas age in the context of repeated infection, they gradually acquire the abilities both to control parasitemia and to resist being clinically ill [1-3]. This gradual acquisition of protection from disease, although not necessarily from infection – and thus referred to as *partial* immunity – is thought to involve both cellular- and antibody-mediated mechanisms. However, a dominant role for anti-malarial antibodies was demonstrated most clearly over five decades ago by the immediate and dramatic clinical and parasitological improvement of Thai children transfused with immunoglobulin from endemic adults [4].

### Field testing functional antibody assays for malaria

Antibody generation has therefore remained an important goal for blood-stage malaria vaccination efforts. Simple anti-repeat region antibodies correlate fairly well with the protection afforded by the most advanced malaria vaccine candidate to date, GlaxoSmithKline’s RTS,S, but the paradoxically consistent presence of individuals who are protected from challenge with barely detectable antibody responses makes this a less than ideal predictive biomarker [5] for this sporozoite-stage vaccine. For blood-stage antigens, although high antibody titers can protect against challenge with the cognate antigens [6], overall titers against the dominant merozoite surface and invasion antigens have correlated poorly with protection from re-infection or disease when measured by simple ELISA [7], making this assay an unreliable surrogate for protection. To date, the only *in vitro* methods to evaluate the functionality of antibodies to the blood-stage parasite are the growth inhibition assay (GIA), which measures the ability of antibodies to slow the expansion of *P. falciparum* parasites cultured in human erythrocytes in the incubator [8], and the antibody-dependent cellular cytotoxicity (ADCC) assay, which has been difficult to generalize to the field [9]. In their current paper, Osier and colleagues [10] have described a new assay, the opsonic phagocytosis assay (OPA), taking advantage of the logical necessity that *in vivo*, responses include not just the antibodies themselves but the other immune cells with which they interact, thus making this assay more biologically relevant. This new functional assay measures the ability of antibodies to opsonize freshly isolated live merozoites for phagocytosis by macrophages in short-term culture. The authors have carefully characterized this assay, and found that it works with both freshly isolated macrophages within lymphocyte mixtures and with an isolated macrophage cell line. It appears readily reproducible to any laboratory with cell and malaria culture capabilities and a flow cytometer.

The most compelling evidence presented by Osier and colleagues are the correlations within the context of two independent longitudinal cohort studies conducted on the coast in Kenya. The naturally acquired responses measured by this OPA correlated with the ability to resist clinical illness in both groups of children residing in this malaria endemic area. Activity in OPA significantly correlated with IgG ELISA but had a weak correlation with GIA (Spearman’s rho −0.358, *P* = 0.041). The addition of this novel functional assay as a biomarker seems a logical step towards a better understanding of the various specificities involved in the development of protective immunity to malaria.

Although the correlation between naturally acquired partial protection and the results of OPA is very suggestive, the link predicting protection against malaria induced by a vaccine presenting a limited number of antigens remains untested. It is possible that a vaccine candidate might induce antibodies that perform well in the OPA, yet fail to predict the outcome of a challenge infection, as has been the case with GIA and anti-malarial antibodies in general. Caution in generalizing to the *in vivo* situation for vaccine development is warranted because of the relatively short transit time (less than 10 minutes) of merozoites between schizont rupture and re-invasion, although the short duration of the assay may functionally mimic this. Because it is an assay that utilizes a whole, living merozoite, its applicability to screen for efficacy of a single-allele, single-antigen vaccine is difficult to determine. Thus, this tool should be championed as a functional assay that, perhaps through future antigen add-back or blocking experiments, may help dissect the diverse antibody responses developing in sequentially exposed individuals to determine which of them are functionally important in resisting disease. This work also sets the stage to develop similar opsonization assays against the infected red blood cell, which is presented to the host immune responses for a considerably longer duration.

## Conclusions

In the headlong race to develop potential malaria vaccine candidates, the as yet undiscovered ‘golden chalice’ remains an *in vitro* assay that can, if not predict the efficacy of a vaccine, at least correlate well with it. One of the perennial frustrations of those seeking to understand the interaction of the malaria parasite with the human immune system is its enormous complexity and the limitations in the tools currently available with which to dissect that complexity. Whether for vaccine development or for understanding the basic immunology that underlies the gradual acquisition of partial resistance to the disease manifestations of malaria parasitemia, the technical advance described in the current paper by Osier *et al*. [10] represents a new tool in the armamentarium of functional immunological assays for malaria.

## Abbreviations

ADCC: Antibody-dependent cellular cytotoxicity
ELISA: Enzyme-linked immunosorbent assay
GIA: Growth inhibition assay
IgG: Immunoglobulin G
OPA: Opsonic phagocytosis assay
